# Hemodynamic Response Imaging: A Potential Tool for the Assessment of Angiogenesis in Brain Tumors

**DOI:** 10.1371/journal.pone.0049416

**Published:** 2012-11-27

**Authors:** Dafna Ben Bashat, Moran Artzi, Haim Ben Ami, Orna Aizenstein, Deborah T. Blumenthal, Felix Bokstein, Benjamin W. Corn, Zvi Ram, Avraham A. Kanner, Biatris Lifschitz-Mercer, Irit Solar, Tsafrir Kolatt, Mika Palmon, Yifat Edrei, Rinat Abramovitch

**Affiliations:** 1 Functional Brain Center, The Wohl Institute for Advanced Imaging, Tel Aviv Sourasky Medical Center, Tel Aviv, Israel; 2 Sackler Faculty of Medicine, Tel-Aviv University, Tel Aviv, Israel; 3 Neuro-Oncology Service, Institute of Radiotherapy, Tel Aviv Sourasky Medical Center, Tel Aviv, Israel; 4 Department of Neurosurgery, Tel Aviv Sourasky Medical Center, Tel Aviv, Israel; 5 Pathology, Diagnostic and Research Cancer Center, Tel Aviv Sourasky Medical Center, Tel Aviv, Israel; 6 Applied Spectral Imaging, Haifa, Hadassah Hebrew University Medical Center, Jerusalem, Israel; 7 Israeli Institute for Advanced Research, Rehovot, Hadassah Hebrew University Medical Center, Jerusalem, Israel; 8 The Goldyne Savad Institute for Gene Therapy, Hadassah Hebrew University Medical Center, Jerusalem, Israel; Johns Hopkins School of Medicine, United States of America

## Abstract

Blood oxygenation level dependence (BOLD) imaging under either hypercapnia or hyperoxia has been used to study neuronal activation and for assessment of various brain pathologies. We evaluated the benefit of a combined protocol of BOLD imaging during both hyperoxic and hypercapnic challenges (termed hemodynamic response imaging (HRI)). Nineteen healthy controls and seven patients with primary brain tumors were included: six with glioblastoma (two newly diagnosed and four with recurrent tumors) and one with atypical-meningioma. Maps of percent signal intensity changes (ΔS) during hyperoxia (carbogen; 95%O2+5%CO2) and hypercapnia (95%air+5%CO2) challenges and vascular reactivity mismatch maps (VRM; voxels that responded to carbogen with reduced/absent response to CO2) were calculated. VRM values were measured in white matter (WM) and gray matter (GM) areas of healthy subjects and used as threshold values in patients. Significantly higher response to carbogen was detected in healthy subjects, compared to hypercapnia, with a GM/WM ratio of 3.8 during both challenges. In patients with newly diagnosed/treatment-naive tumors (n = 3), increased response to carbogen was detected with substantially increased VRM response (compared to threshold values) within and around the tumors. In patients with recurrent tumors, reduced/absent response during both challenges was demonstrated. An additional finding in 2 of 4 patients with recurrent glioblastoma was a negative response during carbogen, distant from tumor location, which may indicate steal effect. In conclusion, the HRI method enables the assessment of blood vessel functionality and reactivity. Reference values from healthy subjects are presented and preliminary results demonstrate the potential of this method to complement perfusion imaging for the detection and follow up of angiogenesis in patients with brain tumors.

## Introduction

Blood vessel visualization and the quantification of different parameters that characterize vessel reactivity and functionality play an important role in the diagnosis and follow-up of several brain pathologies [1]. Previous studies showed high correlations between increased vascularity and tumor malignancy [2], [3]. The complex mechanisms of brain tumor neovascularization formation were recently described. In addition to angiogenesis, vessel co-option and vessel mimicry were also evident in glioblastoma tumors, especially following anti-angiogenic therapies [4]. Yet, noninvasive methods for characterizing tumor vasculature and detecting angiogenesis and other neo-vascularization processes are currently limited.

Magnetic resonance imaging (MRI) is the method of choice for the diagnosis and follow-up of patients with brain lesions. In addition to structural imaging, other techniques provide information regarding brain vascularity and have been increasingly used for clinical decision making. The most common technique is T_1_ weighted (T_1_W) post-contrast enhanced imaging, which identifies areas of disrupted blood brain barrier (BBB) [5]. Correlation with histological findings in patients with gliomas has revealed a direct association between contrast enhancement and tumor neovascularization, endothelial proliferation and cell infiltration [6]. Another commonly used method is dynamic susceptibility contrast (DSC), acquired during contrast agent administration [7]. This method provides information regarding several hemodynamic parameters including cerebral blood volume (CBV) and flow (CBF) [8], and is widely used in a broad range of clinical applications including diagnosis, grading, and assessment of therapeutic response in patients with brain tumors. Additional methods include dynamic contrast enhancement (DCE), which can provide additional information regarding tissue vasculraity and permeability [9], and arterial spin labeling (ASL), which does not require the use of an exogenous contrast agent and provides vascular information mainly regarding CBF [10].

Blood oxygenation level-dependent (BOLD) MRI was originally proposed by Ogawa et al. [11] to study hemodynamic changes related to neuronal activation, and is currently extensively used in functional MRI (fMRI) studies. BOLD imaging was also used in patients with brain tumors during a given task and during rest (resting-state fMRI), to study brain activation and to distinguish tumoral from non-tumoral tissue [12], [13].

BOLD MRI uses deoxyhemoglobin as an endogenous contrast agent, which enables detection of changes in blood flow, volume and oxygenation. Increased BOLD signal can occur due to endogenous effects such as neuronal activity or due to exogenous stimuli such as respiratory challenges of hyperoxia or hypercapnia. Inhalation of pure oxygen causes increased blood oxygenation and reduced blood flow [14], while inhalation of a gas mixture of oxygen with different concentrations of CO_2_ (i.e. carbogen) was shown to increase blood oxygenation and flow. Most studies of brain response to respiratory challenges were based on signal changes in T_2_* imaging [15], [16], [17].

Oxygen inhalation, with or without various concentrations of CO2, has been previously used for several applications: the evaluation of cerebrovascular responses in healthy subjects [18], [19], in patients with brain tumors [15] and in patients with severe carotid stenosis [20]; and for the prediction of tumor response to radiation therapy [17].

Hypercapnic challenge, with brief inhalation of CO_2_ or breath-holding, has also been used in several applications including: assessment of cerebrovascular reactivity in healthy subjects [21], in patients with intracranial stenosis [16] or with cerebral vasculopathy [22]; for understanding the signal mechanism of the BOLD phenomena and for calibrating the fMRI signal [23].

Several animal studies combined both hyperoxia and hypercapnia to distinguish neural from non-neural contributions to fMRI signals [24]; to study changes in the MRI relaxation in brain tumors [25]; to investigate whether breathing a hyperoxic hypercapnic gas mixture could improve the oxygenation of meningiomas [26]; to characterize cerebrovascular responses to both conditions [27]; and to detect mature vessels resistant to anti-angiogenic therapy [28].

The clinical potential of combining hyperoxia and hypercapnia challenges holds great promise. Yet, only a few human studies combine these two conditions for the assessment of brain lesions [29], [30], [31]. During hyperoxia challenge all functional blood vessels are expected to show increased BOLD signal due to changes in blood oxygenation. During hypercapnia challenge, only mature blood vessels, coated with pericyte or smooth muscle cell, will show increased BOLD signal due to vasodilatation, whereas new-immature blood vessels (which form part of the tumor neo-vascularization) will show no changes in BOLD signal. Therefore, by combining these two methods we expect to be able to differentiate between normal and abnormal vascularization, a hallmark indicating pathological vascular conditions such as tumor angiogenesis. This methodology may contribute crucial information to decision-making regarding treatment strategy and for assessment of anti-angiogenic treatment response. The aim of the current study was to evaluate the potential of a protocol combining both hyperoxia (carbogen) and hypercapnia challenges (hemodynamic response imaging (HRI)) for the detection of angiogenesis in patients with brain tumors. For this purpose, reference values were first established in healthy subjects.

## Methods

### Study population

Nineteen healthy subjects (12 women, 7 men; mean age 32.8±11.6 years; mean body weight 69±13 kg) served as a control group. Only subjects with no history of neurological or other diseases and without any brain abnormalities detected by conventional brain imaging were included. The study group comprised seven patients (5 men, 2 women; age range 26–74 years; mean body weight 70±13 kg) with primary brain tumors (six with glioblastoma (GB) - two newly diagnosed and four with recurrent tumors, and one with atypical-meningioma). Patients' clinical data is presented in Table 1. Approval for this study was granted by the local ethics committee in our institute and written informed consent was obtained from all subjects.

**Table 1:** Patients' clinical information.

Patient No	Age (y)	Gender	Diagnosis	Treatment
1	64	F	Atypical Meningioma	Untreated
2	26	M	Glioblastoma	Untreated
3	34	M	Glioblastoma	Untreated
4	74	F	Recurrent Glioblastoma	RT+Chemo
5	45	M	Recurrent Glioblastoma	RT+Chemo
6	58	M	Recurrent Glioblastoma	RT+Chemo
7	41	M	Recurrent Glioblastoma	RT+Chemo

y = years; M/F = male/female; RT = radiation therapy; Chemo = chemotherapy;

### MRI Protocol

MRI scans were performed on a 3T scanner (GE Signa EXCITE HDx, Milwaukee, WI, USA) using a transmit-receive quadrature head coil. The MR protocol consisted of conventional imaging including: 3D inversion recovery spoiled gradient echo sequence T_1_ weighted imaging (WI) (TR/TE/TI = 6.8/1.8/450msec), spin-echo T_2_ WI (TR/TE = 10500/114msec) and fluid attenuation inversion recovery (FLAIR) images (TR/TE/TI = 10002/117/2500msec). All axial slices were aligned along the fourth ventricle and fronto-orbital gyrus. Dynamic susceptibility contrast (DSC) imaging and T_1_ WI post contrast agent injection were performed in all patients. DSC imaging was performed using 2D gradient echo echo-planar imaging (GE-EPI) sequence, acquired alongside administration of Gadolinium-DTPA, with injection rate of 5 ml/sec following by a flush saline. Other imaging parameters were: FOV/matrix = 240 mm/128×128; TR/TE = 1300/30msec, 13 slices with 5 mm thickness with no gap (located at the tumor area) and 78 repetitions.

HRI was performed using GE-EPI sequence with the following parameters: FOV/matrix =   = 240 mm/96x96 (reconstructed to 128×128), TR/TE = 5000/35msec, slices identical to the slices acquired in the conventional imaging sequences. The HRI protocol was applied using a block design paradigm in which subjects inhaled a gas mixture of 95% air + 5% CO_2_ (hypercapnia) or a gas mixture of 95% O_2_ + 5% CO_2_ (hyperoxia, i.e. carbogen), via a standard Hudson face mask, with a rate of 8 pounds per square inch. Initially, optimization of the protocol of both conditions (hyperoxia and hypercapnia) was performed. Based on these results, the final protocol included two separate paradigms starting with HRI-CO_2_ relative to room air serving as baseline (1 min room air, 1.5 min hypercapnia, 2 min room air, 1.5 min hypercapnia, 1 min room air; total of 7 min) followed by HRI-O_2_ (1min room air, 1 min hyperoxia, 1.5 min room air, 1min hyperoxia, 1min room air; total of 5.5 min). The two paradigms were separated in time for at least 10 min during which anatomical images were acquired.

### Data Analysis

#### HRI analysis

Following motion correction, maps of percent signal change (ΔS) during the hyperoxia (ΔS-O_2_, carbogen) and hypercapnia (ΔS-CO_2_) challenges were calculated for each subject, using in-house scripts written in MATLAB and SPM5. The MR signal intensity was correlated to the breathing paradigms (block design analysis), and ΔS values were calculated only for voxels that passed the statistical threshold of *p*<0.05, relative to the first inhalation block (in order to avoid physiological adaptation).

#### Vascular reactivity mismatch (VRM) maps

In order to identify voxels which responded to carbogen but with reduced/absent response to CO_2_, VRM maps were calculated. First, ΔS-O_2_ and ΔS-CO_2_ values were normalized, resulting in 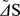-O_2_ and 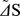-CO_2_ maps, using the following equations:
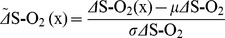


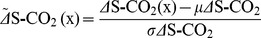
Where: x is the voxel coordinates; ΔS-O_2_/ΔS-CO_2_ are the ΔS values in each voxel on the original maps; µΔS-O_2_/µΔS-CO_2_ are the mean brain ΔS values and σΔSO_2_/σΔSCO_2_ are the standard deviation (SD) of the brain ΔS values. The *µ* and σ values were obtained from the GM and WM areas from both hemispheres in healthy subjects, and from the tumor contra-lateral hemisphere in patients. The GM and WM areas were defined for each healthy subject based on SPM5 segmentation of the HRI-EPI images, by using a probabilistic threshold of 0.8. The ventricle area was excluded using MNI mask. VRM maps were calculated by subtracting the 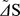-CO_2_ map from the 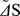-O_2_ map for each subject. The mean and SD values of the VRM in GM and WM of the healthy subjects were measured and served as reference values. In patients, VRM maps were calculated and only voxels above two SD relative to the WM reference value obtained from the healthy subjects were considered as abnormal.

#### Volumes of interest (VOI) analysis

The tumor areas were defined as the enhanced area in the T1W post contrast images for all patients, excluding patient number 2. This patient had a non enhancing tumor and therefore the tumor area was defined as the hyperintense area in FLAIR images. The tumor contra lateral side (CLS) was defined by flipping the tumor VOI (left-right flip). Mean values of relative CBF, ΔS-O_2_ and ΔS-CO_2_ were measured for each VOI. Thresholds were applied to the VRM maps of two SD above mean WM values (for patients number 2–6, having a glial origin lesion), and by two SD above mean GM values (for patient number 1 who had Meningioma). Vascular reactivity index was calculated, representing the number of voxels above the defined threshold (percentage relative to the entire VOI volume).

#### Perfusion analysis

CBV maps were calculated using the perfusion graphical user interface (Penguin) software (www.cfin.au.dk/software). CBV maps were calculated relative to values in the normal appearing WM (rCBV) defined manually in the hemisphere contra lateral to the tumor at the level of the tumor.

All anatomical images and calculated maps were realigned into the MNI152 standard space using FMRIB's linear image registration tool (FLIRT, part of FSL).

#### Histology

Tumor vascularity and vessel-maturation were assessed using histopathological analysis on paraffin-embedded specimens obtained from samples of the tumor tissue (taken from patient number 2 as part of routine surgery protocol). Cell proliferation was assessed by Ki67 immunohistochemistry, tissue vascularity was detected by CD31, and vascular maturation was indicated by anti-SMA. Paraffin sections were immunohistochemically stained using the following antibodies: anti-Ki67 clone SP6 (Thermo Scientific, NeoMarkers), anti-CD31 clone JC70A (Dako) and anti-smooth muscle actin (SMA) clone 1A4 (Dako). Immunohistochemical staining was performed on the Benchmark XT autostainer (Ventana Medical Systems Inc.) using the I-View DAB detection kit (Ventana).

#### Statistical analysis

Statistical analysis was performed on data obtained from the healthy subjects (n = 19) using SPSS 12.0 (SPSS Inc., Chicago, IL, USA). Paired sample t-tests were used to compare the mean ΔS values of the GM and the WM. The mean and SD values of the HRI parameters ( and VRM) obtained from the healthy controls were used as reference values for the patients.

## Results

### HRI in healthy subjects

**Figure 1:**
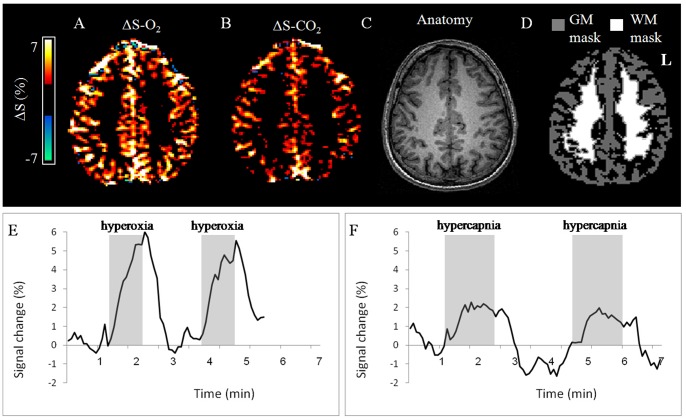
Hemodynamic Response Imaging in the healthy brain. Representative HRI results obtained from a 29 year old healthy subject. (A) ΔS-O_2_ map; (B) ΔS-CO_2_ map; (C) the corresponding T_1_-weighted (anatomical) image; (D) the gray and white matter masks (GM and WM, respectively); the mean time courses of the signal intensity change (%) during hyperoxic challenge (E) and hypecapnic (F) challenges, calculated from the GM VOI of this subject.

Increased MR signal was detected in all healthy subjects during both hyperoxic and hypercapnic challenges in most brain areas. Both ΔS-O_2_ and ΔS-CO_2_ maps showed good separation between GM and WM (Figure 1A,B). The mean ΔS values for both hyperoxic and hypercapnic challenges calculated for GM and WM from all healthy subjects are given in Table 2. Mean ΔS-O_2_ and ΔS-CO_2_ values obtained from the GM were significantly higher compared to those obtained from the WM (*p*<0.001), demonstrating the higher vascularity of this tissue. The brain response to hyperoxic challenge was significantly higher, almost two fold, compared to hypercapnic challenge (*p*<0.002; Figure 1; Table 2).

**Table 2:** Means and standard deviations of HRI values calculated from all healthy subjects.

	GM	WM	GM/WM
ΔS-O_2_	2.54±0.63	0.71±0.30	3.84±1.01
ΔS-CO_2_	1.33±0.33	0.40±0.24	3.69±0.98
VRM	0.50±0.15	0.22±0.10	

ΔS = percent signal intensity changes; GM = gray matter; WM = white matter; VRM = vascular reactivity mismatch.

Following data normalization, distributions of the normalized values (-O_2_ and -CO_2_) in all healthy subjects were similar, yet several brain areas demonstrated positive VRM. These areas were mainly located in the large veins and arteries and in some cortical areas, as identified by a senior neuro-radiologist. The mean VRM values for GM and WM of the healthy subjects, which served as reference values for the patient group, are given in Table 2.

### HRI results in patients

Preliminary HRI results were obtained from seven patients with primary brain tumors, demonstrating the applicability and the clinical potential of the HRI method. Qualitative and quantitative assessment of the ΔS and VRM maps were performed in all patients. Values of ΔS-O_2,_ ΔS-CO_2_, VRM and rCBV of all patients in the tumor and contra-lateral areas are presented in Table 3.

**Table 3:** HRI and rCBV values calculated at the lesion and contra lateral side VOIs.

Patient No		rCBV		ΔS-O_2_ (%)		ΔS-CO_2_(%)		Vascular reactivity index^1^	
		lesion	CLS	lesion	CLS	lesion	CLS	lesion	CLS
1	Atypical Meningioma	8.2	1.9	3.32	2.82	1.09	0.93	19%	9%
2	Glioblastoma	2.5	1.2	1.94	1.93	0.72	1.08	27%	7%
3	Glioblastoma	4.1	NA^2^	1.10	NA^2^	0.33	NA^2^	13%	NA^2^
4	Recurrent Glioblastoma	2.6	1.7	1.00	2.02	0.19	0.60	12%	7%
5	Recurrent Glioblastoma	3.7	1.9	1.05	1.93	0.38	0.87	15%	10%
6	Recurrent Glioblastoma	NA^3^	NA^3^	1.07	1.50	0.63	0.80	2%	12%
7	Recurrent Glioblastoma	NA3	NA3	0.55	1.48	0.09	0.15	13%	43%

ΔS = percent signal intensity changes; rCBV = relative cerebral blood volume; CLS = contralateral side.

1 Vascular reactivity index, number of voxels (in percentages) above the defined VRM threshold.

2 Lesion crosses brain midline, thus contralateral side VOI could not be defined.

3 DSC perfusion was not performed.

#### Patients with newly diagnosed tumors (patients 1–3)

**Figure 2:**
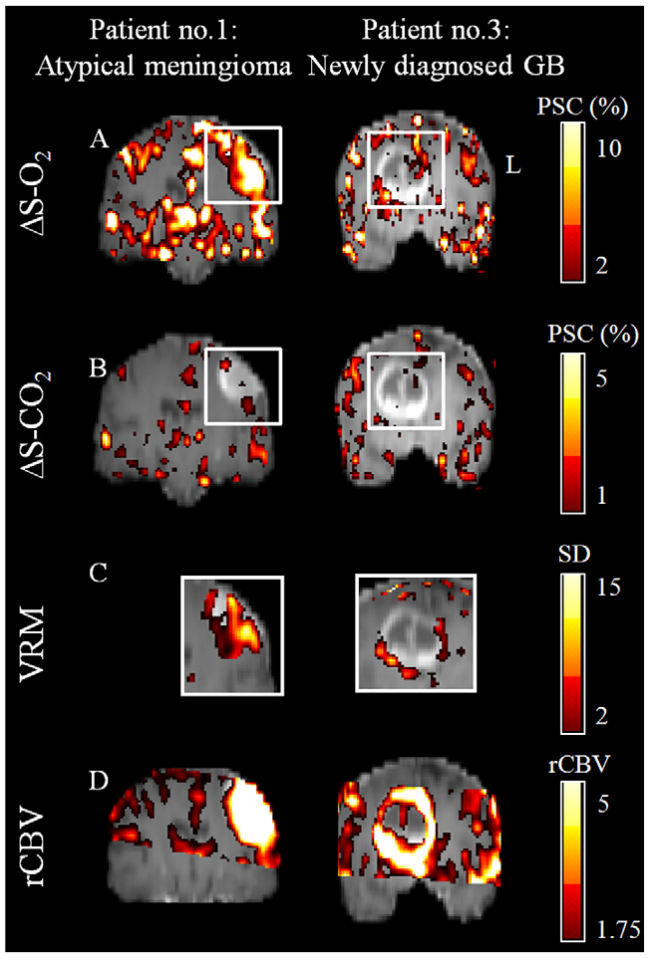
Hemodynamic Response Imaging in patients. HRI results obtained from a patient with atypical meningioma (Left; patient number 1) and from a patient with newly diagnosed glioblastoma (Right; patient number 3); For both patients a representative coronal slice through the tumor center of (A) ΔS-O_2_ maps, (B) ΔS-CO_2_ maps, (C) VRM maps (of the tumors area which are marked by white rectangle on figures A and B) and (D) rCBV maps. All the maps are superimposed on the anatomical images. Color scales for all maps are located on the right side. PSC- percent signal change; SD- standard deviation.

Figure 2 shows results obtained in a patient with atypical meningioma (patient number 1) and a patient with newly diagnosed GB (patient number 3). Increased MR signal was detected in these two patients during hyperoxic challenge, predominantly in the tumor areas (Figure 2A), and to a much lesser extent during hypercapnic challenge (Figure 2B). This divergence is demonstrated on the VRM maps, showing areas that responded to hyperoxia with less response to hypercapnia, within and around these tumors (Figure 2C). In the newly diagnosed GB, a ring-like pattern around the tumor can be observed on the VRM map. High perfusion values (rCBV) were detected in large areas within and around the tumors in these two patients (more than 1.75 fold increase relative to normal appearing WM) (Figure 2D, Table 3). Quantitative assessment of the tumor and CLS areas in patients 1 and 2, showed high VRM values, indicating the abnormal vascularity of these tumors (Table 3). In the patient with meningioma (patient number 1), increased ΔS-O2 value was detected with high VRM value indicating abnormal blood vessels. In the second patient with GB, high response to hyperoxia was observed in both brain hemispheres; however high VRM values were measured only in the tumor. These results may be interpreted as representing tumor angiogenesis. The third patient with newly diagnosed GB in whom no increase in mean HRI values were seen from the entire tumor, had a necrotic area within the tumor, a possible explanation for the obtained values.

**Figure 3:**
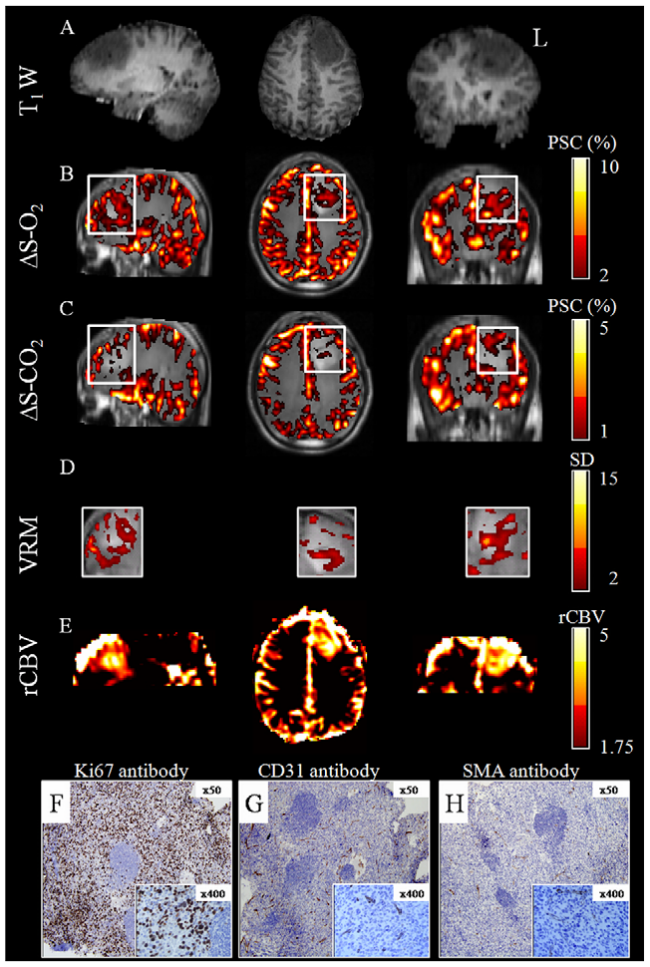
Hemodynamic Response Imaging and pathological findings. Results obtained from a newly diagnosed patient with GB (patient number 2). Representative sagital (left), axial (center) and coronal (right) orientations taken through the tumor center of (A) T_1_-weighted images; (B) ΔS-O_2_ maps; (C) ΔS-CO_2_ maps; (D) VRM maps (of the tumor area marked by white rectangle on figure 3B,C) and (E) rCBV maps. The maps are superimposed on the anatomical images; color scales for all maps are located on the right side. Immunohistochemical staining of samples from the same tumor with: (F) Ki67 antibody for proliferation; (G) CD31 antibody for endothelial cells indicating vascularity and (H) SMA antibody for smooth muscle cells indicating vascular maturation.

Figure 3 shows the imaging results obtained from a 26 year old patient with a newly diagnosed GB (patient 2). In this patient, high vascularity was detected by increased ΔS-O_2_ and high rCBV, even though this was a non-enhancing tumor (with intact blood brain barrier, BBB). The VRM map revealed a ring pattern around the tumor, with high vascular reactivity index value (27%) within the tumor, indicating the presence of new immature blood vessels, Pathological evaluation of samples obtained from this tumor indicated high proliferation index, as demonstrated by Ki67 immunohistochemical staining (Figure 2F); and high vascular density, as shown by anti CD31 immunostaining of endothelial cells (Figure 2G), with only a few SMA positive vessels (Figure 2H).

#### Patients with recurrent GB (patients 4–7)

**Figure 4:**
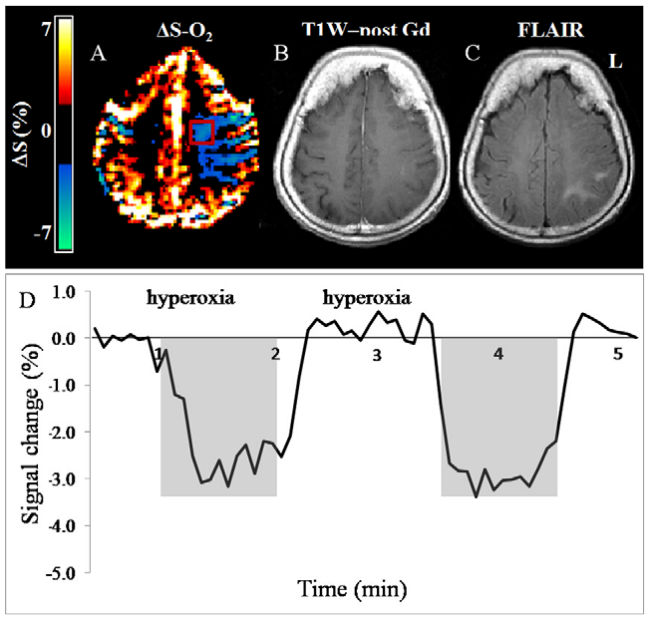
Negative Δ**S-O2 response.** Representative results obtained from a patient with recurrent GB (patient number 4), showing negative ΔS-O2 response during hyperoxic challenge observed distant from the tumor area. (A) ΔS-O_2_ map; (B) T_1_WI post contrast agent injection and (C) fluid attenuation inversion recovery (FLAIR) image. (D) The mean time courses of the signal intensity change (%) during hyperoxic challenge obtained from the area with negative response (VOI is marked by red rectangle on Figure 4A).

In the four patients with recurrent GB previously treated with radiation and chemotherapy, a lower or absent response to both challenges was detected within the tumor and in its surrounding areas. The quantitative results from these tumors and the CLS are given in Table 3. A further notable finding was a prominent negative response during hyperoxia challenge (ΔS-O_2_ <0) detected in two of these patients (patients 4 and 5) in some of the tumor areas and also in normal-appearing brain areas, distant from the tumor location. Figure 4 shows representative data obtained in patient number 4, demonstrating the negative ΔS-O_2_ response in the subcortical WM. Note that these regions were not enhanced in the T_1_WI image obtained post contrast agent injection and showed only mild hyperintense signal on the T_2_ FLAIR images.

## Discussion

In this study, HRI was applied in healthy controls and in patients with primary brain tumors, via a combined protocol of hyperoxic and hypercapnic challenges, in order to indirectly detect the formation of new, immature abnormal blood vessels i.e. angiogenesis. Normal HRI values were obtained from healthy subjects, and used as a reference for the detection of abnormal areas in patients. Results of this study suggest that HRI may provide additional information beyond conventional MR imaging methods regarding vascular reactivity, functionality and maturation and may therefore be useful in the evaluation and follow-up of brain tumors.

The optimized HRI protocol used in this study consisted of two separate scans, each with a block-designed paradigm of either hyperoxic or hypercapnic challenges. A single protocol with alternated blocks of both hypercapnia and hyperoxia was also tested, yet lower signal was detected during hypercapnia when applied after oxygen inhalation. Therefore, when separated into two different protocols the hypercapnic challenge was performed prior to hyperoxia. The two paradigms were distinct in length due to differences in hemodynamic response for each gas [21]. The minimal time interval required between the two gas challenges was defined experimentally to be at least 10 minutes, and was supported by previous findings [32]. In addition, in each paradigm, two blocks of gas inhalation were applied in order to improve the reliability of the statistical maps.

Reference values of normal brain response to hyperoxic and hypercapnic challenges were obtained from healthy subjects. As expected, higher response was detected in the GM compared to WM, reflecting higher vascularity, and enabling a good separation between these tissues. In this study, GM/WM ratios were above 3.5 during both hyperoxic and hypercapnic challenges. Previous studies showed that blood flow is about four times higher in GM compared to WM, whereas blood volume is about two-fold higher in GM compared to WM [33], [34]. Our results from healthy subjects provide combined information about vascular density and blood-flow that might represent vascular reactivity. In the current study, GM and WM VOIs included the entire tissue based on segmentation of the EPI images, thus minimizing partial volume effects.

In patients with GB, abnormal values within the VRM maps were defined only for voxels with 2 SD above the mean WM reference value obtained from all the healthy subjects. Whereas the selection of WM reference is reasonable for tumors of glial origin such as GB, it is less suitable for meningiomas which arise from the arachnoid villi of the meninges. Since there is no ideal reference tissue within the brain for meningioma, for the patient with meningioma, the VRM maps were defined only for voxels with 2 SD above the mean GM reference value obtained from all the healthy subjects. However, other reference values can be used as appropriate in other pathologies.

In patients with newly diagnosed tumors, areas with increased VRM were detected within and around the tumors, likely indicating the presence of angiogenic immature blood vessels that usually characterize high grade brain-tumors. Increased VMR was also detected in the patient with meningioma. This result demonstrates the additional potential application of the HRI method for patients with extra-cranial lesions. The HRI results were validated in one patient with GB using pathological analysis of the tumor. In this patient, a high response during hyperoxia was detected within and around the tumor, indicating areas of high blood vessel density, further supported by anti CD31 immunostaining of endothelial cells. In addition, low response during hypercapnia was detected in this tumor, indicating the inability of the blood vessels to vasodilate, characteristic of immature blood vessels that lack smooth muscle coverage. Pathological findings supported this result with the detection of only focal positivity in SMA staining, as expected in such tumors [35]. Additional studies are needed to validate the HRI results with histological findings using stereotactic biopsy. The usage of a combined hyperoxic and hypercapnic challenge and the calculated VRM maps enables monitoring of the maturation status of the tumor blood vessels. This information, which currently can only be obtained from histological evaluation, may contribute significantly to the diagnosis and grading of brain tumors.

A reduced or absent vascular reactivity to both challenges was observed in all patients with recurrent GB, who had previously undergone aggressive treatment including multiple surgeries, radiation and chemotherapies. This pattern may indicate either the direct therapy effects on blood vessels or the effect of tumor progression, resulting in vasogenic edema leading to pressure on the vasculature, both of which may affect vessel reactivity. These findings indicate that the use of the HRI method may be less suitable in patients who have already undergone treatment, yet future studies are needed to assess the use of HRI method to monitor changes following anti-angiogenic therapies which are expected to “normalize” the abnormal tumor blood vessels [36].

Furthermore, in two patients with recurrent GB, large areas with a strong negative response during hyperoxia, and a lack of response during hypercapnia were detected both adjacent to, and distant from the tumors. This phenomenon may be related to “intracerebral steal” effect, attributed to shunting of blood flow away from non-autoregulating ischemic areas by the action of normally reactive vessels in an attempt to increase the flow to ischemic areas [37]. The use of carbogen inhalation, which contains 5% CO_2_, may induce a similar effect, resulting in reduced MR signal. GB tumors are usually heterogenic with ischemic areas that may also cause “intracerebral steal”. Further studies should test this hypothesis and determine whether these changes are reversible following anti-angiogenic therapy, and whether this finding may predict therapy response.

The strength of this study is the demonstration of the feasibility of the HRI method for indirect structural and functional imaging of brain vascularity. Currently, characterization of vascular tissue properties, and indication of the presence of angiogenesis in patients, can be obtained only based on pathological findings. Thus, non-invasive imaging methods that can provide similar information is of great clinical importance. The combination of HRI with other advanced MRI methods, such as DSC and dynamic contrast enhanced imaging, which provide different vascular features, may assist in tumor diagnosis and follow-up. Contrast-enhanced imaging is the most commonly used technique in brain tumor imaging, representing the BBB breakdown. However, this method is non-specific and lacks sensitivity, particularly in infiltrative high grade gliomas [2]. Although contrast-enhancement usually correlates with tumor malignancy, some high grade tumors do not enhance with contrast agents [38] as was observed in patient number 2. Another common method for brain tumor diagnosis and follow-up is DSC imaging [7], [39]. This method provides information regarding blood vessel density and flow; however, it necessitates intravenous access and contrast agent injection. This is not a trivial matter for populations such as pediatric groups, patients who have received extensive chemotherapy, pregnant women and patients with renal failure. Another limitation of this method is that it only covers partial brain volumes due to the necessity of high temporal resolution. HRI, as shown in this study, provides functional dynamic information from the entire brain without the need for contrast agent administration, and can thus be used in patients who have contraindications.

## Conclusions

This study demonstrates the applicability of the combined hyperoxic and hypercapnic fMRI protocol in healthy subjects and in patients with primary brain tumors. Preliminary results in these patients show that HRI, correlating with pathological findings, may provide complementary vascular information to the DSC method and in combination with additional structural and functional information (such as diffusion weighted imaging) may provide a more comprehensive assessment. Further large-scale studies correlating between HRI and pathological findings are needed in order to better understand and interpret the HRI contrast in normal and pathological brain lesions, and to assess the clinical value of this method in the diagnosis and grading of brain tumors. Future applications may include follow-up of patients with brain tumors receiving anti-angiogenic therapy, early prediction of radiotherapy response and assessment of vascular reactivity to predict stroke risk.
